# Challenges in implementing uncomplicated malaria treatment in children: a health facility survey in rural Malawi

**DOI:** 10.1186/s12936-017-2066-7

**Published:** 2017-10-18

**Authors:** Alinune N. Kabaghe, Mphatso D. Phiri, Kamija S. Phiri, Michèle van Vugt

**Affiliations:** 1 Center of Tropical Medicine and Travel Medicine, Department of Infectious Diseases, Division of Internal Medicine, Academic Medical Center, University of Amsterdam, Meibergdreef 9, PO Box 22700, 1100 DE Amsterdam, The Netherlands; 2 Public Health Department, College of Medicine, Private Bag 360, Blantyre, Malawi; 3 Ministry of Health, Queen Elizabeth Central Hospital, P.O. Box 95, Blantyre, Malawi; 4 Malawi-Liverpool Wellcome Trust, P.O Box 30096, Blantyre, Malawi

**Keywords:** Malaria, Health systems, Clinical decision making, Care-seeking

## Abstract

**Background:**

Prompt and effective malaria treatment are key in reducing transmission, disease severity and mortality. With the current scale-up of artemisinin-based combination therapy (ACT) coverage, there is need to focus on challenges affecting implementation of the intervention. Routine indicators focus on utilization and coverage, neglecting implementation quality. A health system in rural Malawi was assessed for uncomplicated malaria treatment implementation in children.

**Methods:**

A cross-sectional health facility survey was conducted in six health centres around the Majete Wildlife Reserve in Chikwawa district using a health system effectiveness approach to assess uncomplicated malaria treatment implementation. Interviews with health facility personnel and exit interviews with guardians of 120 children under 5 years were conducted.

**Results:**

Health workers appropriately prescribed an ACT and did not prescribe an ACT to 73% (95% CI 63–84%) of malaria rapid diagnostic test (RDT) positive and 98% (95% CI 96–100%) RDT negative children, respectively. However, 24% (95% CI 13–37%) of children receiving artemisinin–lumefantrine had an inappropriate dose by weight. Health facility findings included inadequate number of personnel (average: 2.1 health workers per 10,000 population), anti-malarial drug stock-outs or not supplied, and inconsistent health information records. Guardians of 59% (95% CI 51–69%) of children presented within 24 h of onset of child’s symptoms.

**Conclusion:**

The survey presents an approach for assessing treatment effectiveness, highlighting bottlenecks which coverage indicators are incapable of detecting, and which may reduce quality and effectiveness of malaria treatment. Health service provider practices in prescribing and dosing anti-malarial drugs, due to drug stock-outs or high patient load, risk development of drug resistance, treatment failure and exposure to adverse effects.

## Background

Significant malaria burden reduction in high transmission areas will strongly rely on high community utilization, and effective coverage of health interventions by health systems [1]. Prompt and effective malaria case treatment, with artemisinin combination therapy (ACT), is key to reducing morbidity and mortality and advocated by World Health Organization (WHO) [2, 3]. Apart from prompt diagnosis and treatment, the WHO also recommends rational use of anti-malarial agents, and appropriate weight-based dosing [3]. The use of ACT reduces malaria transmission by reducing sexual parasites hence lowering patient infectiousness [4–6]. Delays in seeking care, and poor quality management of malaria drive infection transmission. High parasitaemia from delayed care-seeking and ineffective treatment are both associated with increasing gametocytaemia which increases human to mosquito infection and hence transmission [7, 8]. With the current scale up of ACT coverage, and declining disease burden [9, 10], there is need to ensure maximum treatment effectiveness i.e. high quality case management and treatment adherence, and good patient and guardian health-seeking behaviour [11].

Sub Saharan Africa, where 88 and 90% of all malaria cases and deaths occurred in 2015 respectively, has weak health systems producing less than the expected positive health outcomes [9, 12]. In Malawi, as in most of the sub-Saharan region, district health systems oversee all health services through the District Health Office (DHO). The DHO is responsible for ensuring maximum coverage and quality assurance of all health services.

**Fig. 1:**
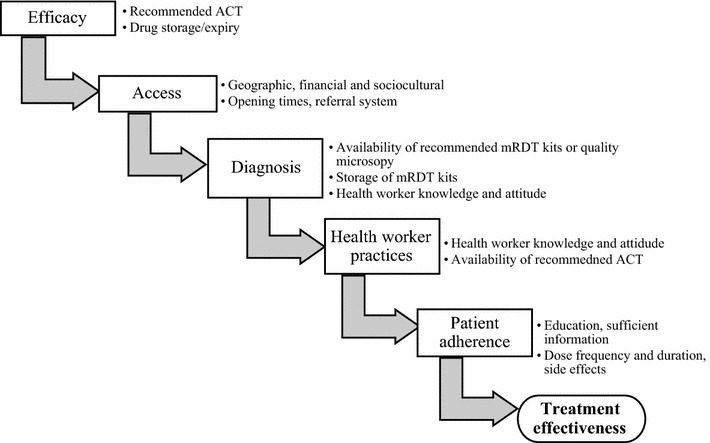
Systems effectiveness framework summarising health system-, health service provider-, and patient-factors affecting malaria treatment effectiveness. The flow diagram shows the five important steps, with examples of factors which affect their implementation, for prompt and effectiveness diagnosis and treatment of malaria. *ACT* artemisinin-based combination therapy, *mRDT* malaria rapid diagnostic test (Adapted from MALERA consultative group [1])

Treatment implementation has five critical steps (Fig. 1): efficacy, access, diagnosis, health service provider practice, and patient adherence [1, 13]. Several factors affect implementation at each step, falling into one of the following broader categories: health system, health service provider, and patient [14, 15]. Low indicators at any step present a bottleneck to the overall performance of the system. Diagnosis and health service provider practices include: availability of appropriate diagnostic test; failure to request for a malaria test; non-adherence to the malaria test results; prescribing low quality ineffective drugs; prescribing of non-recommended anti-malarial drugs [13, 16, 17]. Patient factors, such as beliefs and education level affect access and promptness to seek treatment as well as adherence to treatment schedules and dose [18–20]. To ensure maximum effectiveness, performance at each critical step should be assessed and maximized. Available tools, such as health facility reports, are inconsistent and incomplete, and are inefficient for assessing quality of implementation at individual level [21–25].

A health facility survey was conducted to assess implementation of uncomplicated malaria treatment for children aged 0–5 years old with uncomplicated malaria in rural Chikwawa district in Malawi. The survey focused on: availability of efficacious treatment, access to treatment services including promptness to seek care, diagnosis of malaria, and health provider’s practices. An assessment of the existing patient registration and reporting system in monitoring implementation quality of treatment was also conducted.

## Methods

### Study design

**Fig. 2:**
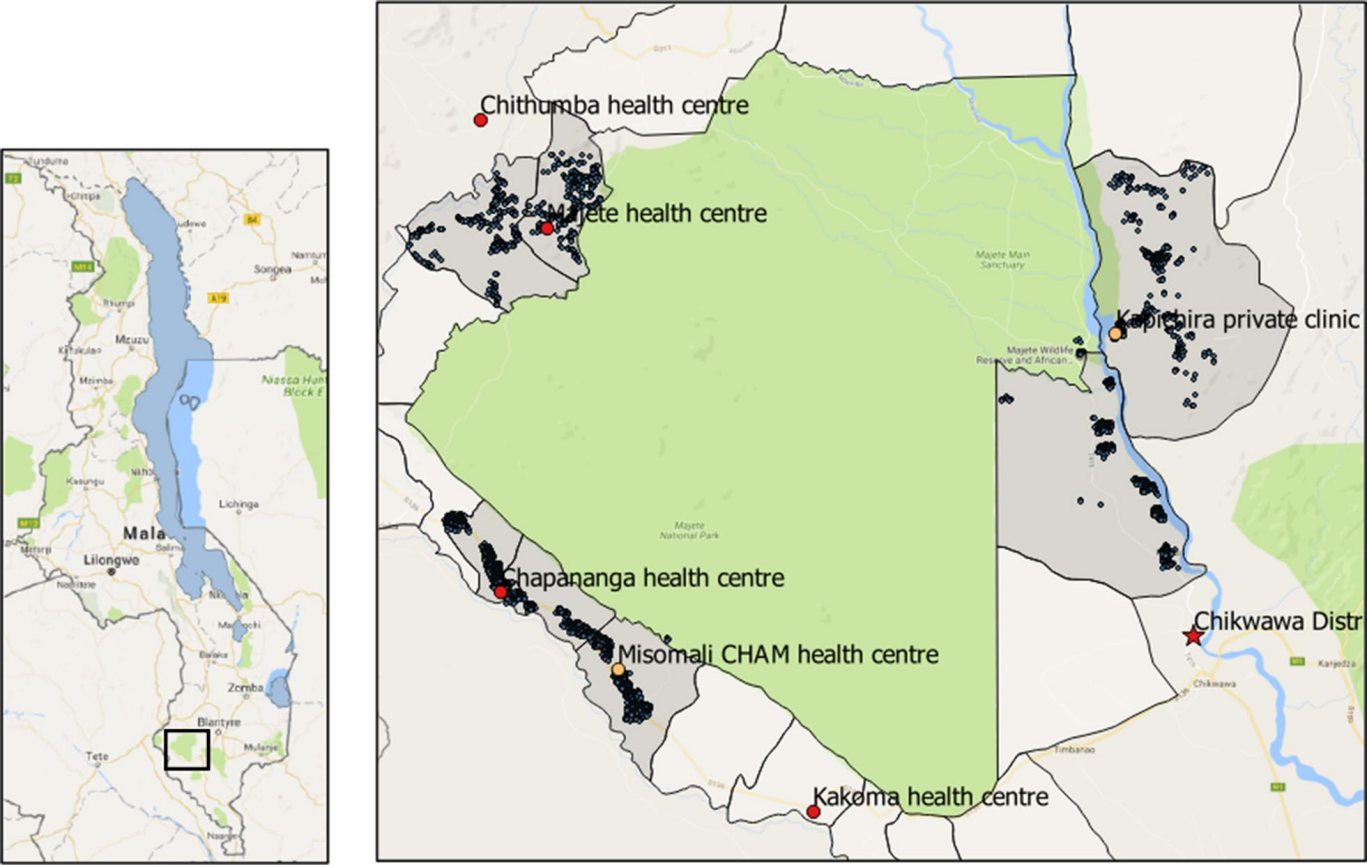
Map of health centres around the Majete Wildlife Reserve Perimeter. On the left, the map of Malawi showing the location of Majete Wildlife Reserve in the South (rectangle); on the right, the surrounding health centres (red and orange circles for government and private facities respectively), the district hospital (red star) and all households (blue circles) in Majete Malaria Project catchment area (grey)

A cross sectional survey was conducted in primary health care facilities surrounding the Majete Wildlife Reserve in Chikwawa district in southern Malawi. The facilities were chosen purposively under the Majete Malaria Project (MMP) catchment area (Fig. 2).

### Study setting

The survey was conducted within the MMP catchment area in Chikwawa district, southern Malawi from December 2015 to May 2016. Chikwawa district is located within the East African Great Rift valley and experiences frequent flooding of its main river, Shire, during the rainy season. The rainy season, occurring from December to May leads to peaking of malaria transmission. The main project focuses on communities surrounding the Majete Wildlife Reserve with a projected total population of approximately 150,000 (African Parks, 2014). MMP aims to reduce malaria burden in this area through a behavioural change communication approach implemented by trained community volunteers.

The surveys were conducted in health-care facilities, known as health centres, which are either public (funded by government), or non-public (funded by non-governmental institutions, individuals or religious organizations). The health centres provide basic primary health care including diagnosis and treatment of uncomplicated malaria. Patients presenting at a health centre, requiring hospital admission or surgical procedures, are referred to the district hospital for secondary health care. The health centres are staffed by at least one medical assistant or clinical officer, a nurse-midwife technician, and community health workers known as Health Surveillance Assistant (HSA). Medical assistants and clinical officers are a cadre of clinicians with an 18-month certificate and 3-year diploma training in clinical medicine, respectively; nurse-midwife technicians possess a diploma in nursing and midwifery following 3-year training. HSAs have basic disease surveillance training and on the job training in programmes such as immunization, community case management including treatment of malaria, HIV counselling and testing, etc. Some HSAs are based at the facility while others are in the community providing assorted basic primary care such as immunization of children, non-invasive family planning methods, and integrated management of childhood illnesses (IMCI) in village clinics. The Malawi Ministry of Health recommend one HSA for every 1000 population.

For uncomplicated malaria diagnosis, the national treatment guidelines [26] recommend confirmation of cases using a malaria Rapid Diagnostic Test (recommended histidine-rich protein-2 (HRP 2) products: *SD Bioline malaria Ag Pf* or *Paracheck malaria Ag Pf*) or microscopy. The first-line treatment for confirmed uncomplicated malaria in children above 5 kg body weight and pregnant women in the second and third trimester is artemether–lumefantrine (AL); for children less than 2 months or less than 5 kg body weight and pregnant women in the first trimester, a combination of clindamycin and quinine is recommended. Sulfadoxine–pyrimethamine (SP) and artesunate–amodiaquine (ASAQ) are reserved for intermittent preventive treatment in pregnancy and uncomplicated malaria treatment failure, respectively. Testing with RDT and treatment with AL are free in both public and non-public facilities as government provides test kits and drugs. Non-public facilities may charge a small consultation fee or require individuals to pay for non-malarial drugs, such as painkillers. During the surveys, village clinics were providing uncomplicated malaria treatment to children below 5 years old based only on fever or history of fever without a confirmatory test (although plans were underway to make confirmatory testing mandatory in village clinics).

### Data collection and analysis

Enumeration of households in the main project’s catchment area was conducted in the area prior to data collection from August to November 2014. Geolocations of all health centres in the Majete Wildlife Reserve perimeter were collected using Global Positioning System (GPS) devices on Samsung Galaxy Tab 3 running Android 4.1 Jellybean Operating System, on open data kit (ODK) platform (Fig. 2).

Data were collected by two qualified medical doctors using a structured questionnaires administered to the health facility personnel in-charges, guardians of eligible children, and health management information systems (HMIS) officer based at DHO. The health facility in-charges were interviewed on availability and cadre of health personnel, medical supplies and equipment, and services provided. The focus for medical supplies was availability of AL and RDT kits. AL is packaged in blister packs containing six fixed-doses for one treatment course per body-weight category as follows: one tablet for 5–14.9 kg; two tablets for 15–24.9 kg; three tablets for 25–34.9 kg; and four tablets for the above 35 kg category. Availability of these blisters and RDT kits, was confirmed with records on stock-cards and drug store. Availability of equipment included weighing scales, thermometers, and glucose and haemoglobin monitors. The study team observed and recorded patient flow at each health facility. The HMIS officer, based at the DHO, was interviewed on the structure and reporting of health information and its functionality. The officer is responsible for receiving all reports from all health facilities in the district and ensuring it is sent to a web-based, District Health Information System (DHIS2).

For the exit interviews, all children aged 5 years old and below, presenting at the health facility on any of the 3 working days the study team spent at the facility, were eligible for participation. Children and their guardians were consecutively referred to the study team by the health service provider after receiving all treatment. Children requiring surgical attention, or severely ill, or attending special clinics were excluded. The health workers were only informed that the participants would be interviewed about MMP project and reassessed for another study; the main purpose of the survey was not disclosed to the health workers.

Socio-demographic information including child’s age, presenting symptoms and their duration, and past medical treatment were obtained from the guardian during the interview. The reported time taken travelling from home to the health facility was also recorded for a few guardians. Weight and temperature were re-measured using a calibrated analogue weighing scale and digital electronic thermometer, respectively. Details of care, provided during that visit, including malaria RDT results, and drugs and doses prescribed, were captured from the participant health passport book—a record of a person’s health care-related information. The children were not retested for malaria. All data were entered into an electronic case report form on a Samsung Galaxy™ tablet through the ODK platform.

A checklist following Malawi treatment guidelines for management of malaria [26] was used to assess prescriber’s adherence to guidelines. Definition of the following terms were based on the guidelines:

1. Uncomplicated malaria: was a fever or history of fever within the preceding 24 h and a positive RDT result. Children with any sign or symptom of severe malaria, or history of malaria treatment within the preceding 14 days were excluded.
2. Adherence to guidelines included: testing all febrile children with malaria RDT; prescribing AL to RDT positive patients and not prescribing AL to RDT negative patients.
3. Correct AL dose: was the dose of AL prescribed based on the appropriate weight category of the child.

SPSS version 22 [27] was used to analyse quantitative data. QGIS version 2.8.2 (QGIS Development Team, 2015—Open Source Geospatial Foundation) was used to map straight line distance between furthest households to a health facility.

After data collection and analysis, the findings were disseminated to the health facility personnel, i.e. nurses and clinicians, and the District Health Management Team (DHMT). DHMT was comprised of the District Health Officer, District Nursing Officer (in charge of district nursing services), District Medical Officer (in charge of district clinical services), and malaria coordinator (in charge of all malaria related activities in the district). A formal discussion, henceforth “dissemination discussion”, on the survey findings, including factors affecting health care practices, was conducted; feedback from the discussion is also reported in this paper.

### Ethical consideration

The study was reviewed and approved by College of Medicine Research and Ethics Committee (P.05/14/1579). A written informed consent was sought from guardians of eligible children. Chikhwawa District Health Office authorised the approval to be conducted in the district.

## Results

Six health centres in southern Malawi, namely Kakoma, Kapichira, Chithumba, Majete, Misomali and Chapananga, were visited (Table 1), with a combined catchment population of 71,383 people; 120 exit interviews were completed. Kakoma health centre had the highest catchment population (22,725) while Kapichira had the lowest with 5945. Chithumba health centre had the most number of children sampled, while Kakoma and Kapichira had the least people.

**Table 1:** Summary of health facility characteristics

Ownership	Chapananga	Chithumba	Kakoma	Kapichira	Majete	Misomali	Total
	Govt.^a^	Govt.	Govt.	Private	Govt.	Private	
Catchment population	16,783	10,755	22,725	5945	6000	9175	71,383
Health service providers^b^	3	2	2	3	3	2	15
Health service provider per 10,000	1.8	1.9	0.9	5.0	5.0	2.2	2.1
HSA^c^	9	7	13	7	2	4	42
Population per HSA^d^	1865	1536	1748	849	3000	2294	1680
Children sampled	23	40	12	12	20	13	120

^a^Government owned

^b^Health service provider refers to a clinician or nurse who prescribed anti-malarial drugs

^c^Health Surveillance Assistant

^d^Ministry of Health recommends one HSA per 1000 people

Each health centre had 2–3 health service providers, where a health service provider is a nurse or clinician. The average health service provider-to-population ratio was 2.1 per 10,000 population with the lowest in Kakoma (0.9 per 10,000) and highest in Kapichira (5.0 per 10,000). Number of HSAs varied per health facility from 2 to 13. Only Kapichira health centre had Ministry of Health requirement of one HSA per 1000 population (849) while Majete had the highest population per HSA (3000).

For the exit interviews (Table 2), the median, mean age of the children was 1.7 years old which was mainly in the 0–1 and 0–2 age categories; 53.3% were males. Most of the participants came with their mothers as guardians. Two-thirds of the guardians had attained some primary school education, although 10.8% of guardians reported no formal education at all.

**Table 2:** Summary of demographic characteristics of children and guardians in the exit interviews

Characteristic	Frequency	%
Total children	120	
Median age in years (IQR)	1.7 (0.9–3.2)	
Age categories in years		
0–1	35	29.2
1–2	35	29.2
2–3	18	15.0
> 3	32	26.7
Male	64	53.3
Confirmed fever^a^ (temperature > 37.5 °C)	61	50.8
Type of guardian		
Mother	108	89.2
Father	11	9.2
Other	2	0.8
Education level of guardian		
None	13	10.8
1–4 years of primary	80	66.7
5–8 years of primary	12	10.0
Any secondary	15	12.5

*IQR* interquartile range

^a^Temperature as measured by study personnel

### Availability of efficacious treatment

During the survey period, one health centre reported complete stock-out of all AL blister-packs in the preceding 3 months of the survey. There were AL stock-outs of one or more, but not all, blister-packs in four of the six remaining facilities in the same period. During the dissemination discussion, late and inaccurate reporting of anti-malarial supplies were identified as contributing to the stock-outs; drug pilferage and overprescribing of anti-malarial drugs were cited as other potential factors.

### Access and care-seeking of services

The minimum straight-line distance required for a guardian and child to access health services was 2.5 km and the maximum was 14.7 km. Most of the population on the eastern side of the reserve were located further from a health facility compared to the western side (Fig. 2).

Guardians of 59% (95% CI 51–69%), 23% (95% CI 14–32%) and 18% (95% CI 10–28%) of children reported the duration of symptoms as less than 24, 24–48 h and more than 48 h, respectively, before presenting to a health facility. Forty-seven of interviews recorded the approximate time taken to reach the health facility; most participants travelled less than 1 h to reach the facility (51.0%) although others reported travelling more than 2 h (12.8%).

### Diagnosis and medical equipment

At the time of the survey, all facilities had malaria RDT kits and reported no stock-outs in the preceding 3 months of the survey; this information was also confirmed from the drug store and stock cards. Easy to use point of care diagnostic devices, such as hemocues and glucometers, are ideal in primary health care facilities. Only Kapichira health centre was checking haemoglobin (using HemoCue 201). None of the health centres had glucometers for determining blood glucose levels. All facilities had functional infant and adult weighing scales, thermometres and height boards.

### Health providers’ practice

A hard copy of national malaria treatment guidelines was available in all the health centres. However, only 34 children (28.3%) had their weight measured and recorded by health facility staff, and 103 (85.8%) had a recorded date of birth. Seventeen percent (n = 21) of the children had a temperature measurement recorded by health centre staff. When temperature was rechecked at exit, 61 children (50.8%) had an axillary temperature above 37.5 °C.

During the results dissemination discussion, health facility personnel cited inadequate number of personnel, leading to high workload, as contributing to not measuring and/or recording weight and temperature.

**Fig. 3:**
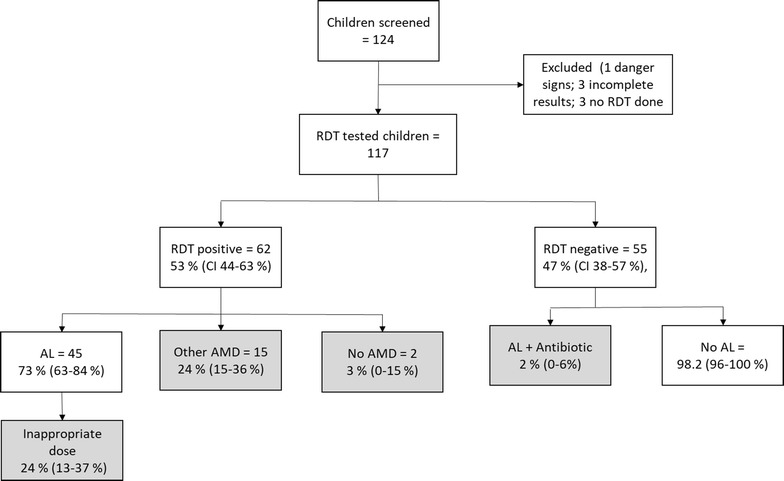
Flow chart for enrolment of participants, mRDT results, and treatment provided. Number of patients inappropriately managed according to the guidelines are shown in the grey shaded boxes. Numbers in parentheses are 95% confidence interval of the proportions. *AMD* antimalarial drug, *AL* artemether–lumefantrine, *mRDT* malaria rapid diagnostic test, *SP* sulfadoxine–pyrimethamine

Malaria RDT was administered to 117 of 120 (97.5%) eligible children (Fig. 3). RDT results were positive and negative in 53% (95% CI 44–63%) and 47% (95% CI 38–57%), respectively, of the children.. Adherence to not prescribing AL to negative RDT results was 98% (95% CI 96–100%) compared to 73% (95% CI 63–84%) of RDT positive children who received the recommended first-line treatment, AL. Of the AL recipients (n = 45), 11 were prescribed at least one antibiotic. The remaining RDT positive participants received other (non-recommended) antimalarial drugs: ASAQ, SP and quinine; 3% (0–15%) RDT positive patients did not receive an anti-malarial drug. Antibiotics were prescribed to 17% (95% CI 10–27%) and 80% (71–91%) of children with malaria RDT positive and RDT negative, respectively. During dissemination discussion, personnel reported prescribing non-recommended anti-malarial drugs due to stock-out of the recommended first-line drug AL.

Based on weight category, 24% (95% CI 13–37%) of the children who received AL were inappropriately dosed; 2% (95% CI 0–14) were under dosed and 22% (95% CI 11-–34%) were over dosed. Comparing age- and weight-based dose categories, 31% (20–46%) of the children in the 3–9 year age group (2 tablets) had a weight of less than 15 kg (1 tablet). to the 1-tablet category by weight, were in the 2-tablet category based on their age.

### Health information systems

**Fig. 4:**
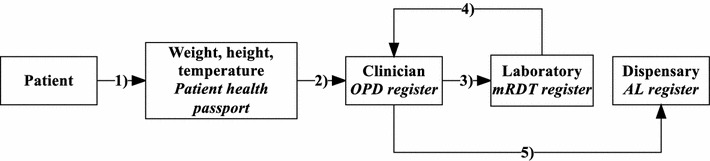
Patient flow and information capture in the health facilities. The numbers are the sequence of patient flow beginning with (1) measurement of weight, height and temperature which are recorded the patient’s health passport, (2) health worker assessment, (3) laboratory investigation—an mRDT for malaria; in the laboratory, the name, age, village of origin and mRDT result of the patient are recorded in the *mRDT register*, (4) the health worker makes a diagnosis and prescribes drugs, the HMIS officer records the name, age, diagnosis and treatment given to the patient in the *OPD register*, (5) the patient receives drugs from the dispensary where the name, age and AL dose prescribed are recorded in the *AL register*. *AL* artemether–lumefantrin, *OPD* outpatient department, *mRDT* malaria rapid diagnostic test

Patient information was recorded in three registers: malaria RDT results, AL and outpatient registers (Fig. 4). All patients presenting at the facility were required to have their weight, height and temperature measured; they were then assessed by a health service provider (clinician or nurse) who, after clinical assessment, ordered a malaria test in the laboratory; the results of the test were to be recorded in the RDT register together with the name, age and village of the patient; the patient was then to be sent back to the health service provider with the RDT result; the provider was required to make a diagnosis based on the result, record the diagnosis in the outpatient register with the name age and village of patient, and prescribe drugs. If the patient was prescribed AL, this was to be collected at the drug dispensary and recorded in the AL register. An HMIS clerk then has to record name, age and village of patient and the diagnosis and treatment in the outpatient register. Incomplete AL registers were found in two facilities while; in one facility, the register was not used at all. There were inconsistencies between the different registers; patients were recorded in one register but missing from the others. During the dissemination discussion, staff reported high workload as a factor contributing to incomplete records.

Each month, a report is supposed to be sent to the DHO summarizing the number of malaria RDTs performed, number of malaria cases diagnosed and AL doses consumed. Number of malaria cases diagnosed are to be reported in two age groups: under 5 and over 5 years old. An HMIS officer at the DHO enters this data into a web-based server, the District Health Information System (DHIS) [28]. Programme coordinators and senior DHO personnel can access this data and generate summaries.

## Discussion

This survey presents a practical approach for assessing factors affecting implementation of uncomplicated malaria treatment in children in a rural setting with added value of immediate feedback. Based on the systems effectiveness framework assessment, the survey has identified health worker practices, health information system, and drug availability as critical bottlenecks affecting the implementation and effectiveness of uncomplicated malaria treatment in children aged 0–5 years. Health worker practices including prescribing inappropriate drug dose, prescribing non-recommended anti-malarial drugs, and overprescribing of antibiotics promote emergence of drug resistance [29], prevent clearing of parasite due to insufficient drug plasma concentrations [30], and increase the risk of adverse events. Adverse events influence patient and community beliefs, hence their treatment adherence. Drug resistance and failure to clear parasites both affect treatment effectiveness. Routine monitoring and evaluation tools focus on coverage and service utilization i.e. numbers accessing the service, and neglect the quality of the intervention which requires emphasis [31].

The survey showed that most health workers prescribed anti-malarial drugs based on age than weight which is consistent with previous findings [29]. Relying on age-based dosing in resource-limited settings may lead to inaccurate dosing in children with malnutrition which often coexists with malaria. Differences in classifying AL dose based on age than weight were evident in the study. This discrepancy highlights the significance of low weight-for-age in the survey setting. Health workers attributed high work load as the reason for not weighing most of the patients. From the survey, all facilities had a low number of health service providers compared to the respective catchment population hence high patient load which may have compromised the quality of patient care as reported in other studies [32]. On the guardian side, low level of education reported in this survey may also have affected the accuracy of the children’s age in the absence of documented date of birth.

A high proportion of malaria RDT positive febrile children were prescribed antibiotics in this study similar to studies in Uganda and Zambia [33, 34]. This practice can be beneficial or inappropriate in malaria endemic regions. Malaria parasitaemia in individuals in endemic areas, who develop some immunity, may or may not be the cause of fever or other symptoms of illness. In asymptomatic parasitaemia, covering the underlying cause of the symptoms is beneficial while also clearing the parasite with an anti-malarial will reduce the reservoir of malaria infection. Covering for a “presumed” bacterial infection in a symptomatic malaria patient is wasteful of antibiotics and promotes drug resistance. Febrile disease diagnosis still needs to improve for both RDT positive and negative febrile patients to guide prescribers on appropriateness of antibiotic treatment [13, 35].

Though more than half the guardians reported duration of symptoms before seeking treatment as 24 h, up to 40.1% presented after 24 h, slightly lower than other study findings [36, 37]; worryingly, 18.3% presented after 48 h of symptoms. Although the sample was not powered to evaluate factors associated with delays in seeking care, some of the findings in the survey such as low level of education, distance to facility, and rural communities have been reported as significant contributors to care-seeking behaviour [38–41]. Delay in treatment increases the risks of morbidity and mortality, and propagates malaria transmission as the patient remains infectious for a longer period of time. In this survey, barriers to prompt care-seeking were not qualitatively investigated though previous studies in the same geographic region reported distance, long waiting time and poor health worker attitude as community barriers to seeking care [42].

Health information registers were unreliable in some of the facilities—as shown in other studies in sub-Saharan Africa [21, 22]. Late submission of malaria data reports to the district health office further affected the utility of health information which is required for drug ordering and supply. These practices contributed to drug stock-outs leading to prescription of non-recommended anti-malarial drugs.

This study had a number of limitations. Although the survey was done in few health facilities, a comprehensive investigation in the small study region highlights bottlenecks in implementing uncomplicated malaria treatment applicable in larger settings and for other diseases. The survey did not assess patient adherence to treatment, a key component in treatment effectiveness [1, 13, 43]. Factors influencing promptness to seek care were not explored qualitatively in this study as well as health worker factors influencing clinical practice though some factors were cited during the result discussion session with the health facility personnel and district health management team.

With WHO’s core principles of early diagnosis and effective malaria treatment, use of ACT, appropriate anti-malarial use and appropriate drug dosing by weight [3], the survey highlights, at a small scale, inappropriate prescribing practices and delays in care-seeking with far-reaching implications at a large scale. The findings may apply in other health facilities and communities in Malawi and sub-Saharan Africa. A robust system, to routinely monitor and evaluate implementation and quality of management of patients, is required not only for malaria, but also for other diseases. The current reports which aggregate data based on two age categories, under- and over-5 years, do not capture the appropriate AL doses consumed nor the appropriateness of the dosing.

## Conclusion

The study highlights both community and health system factors which may affect uncomplicated malaria management. By using patient exit interviews the survey highlights areas of service delivery which may not be detected with routine facility surveillance. Inappropriate health worker’s prescribing practice may promote development of drug resistance to first line anti-malarial drugs, treatment failure, and increase risk of adverse events. There is therefore a need to monitor the quality of service delivery to avoid compromising its effectiveness through such surveys. Incorporating other health system factors during the evaluation may help to identify and address the factors. For example, strengthening the health management information systems may reduce stock-outs and improve clinical practice for uncomplicated malaria management. Prompt care-seeking for fever was low and will require community engagement strategies to improve.

## Abbreviations

ACT: artemisinin-based combination therapy
AL: artemether–lumefantrine
ASAQ: artesunate–amodiaquine
COMREC: College of Medicine Research and Ethics Committee
DHIS: District Health Information System
DHMT: District Health Management Team
DHO: District Health Office or District Health Officer
GPS: Geographic Positioning System
HMIS: Health Management Information System
HRP: histidine-rich protein
HSA: health surveillance assistant
IMCI: integrated management of childhood illnesses
IQR: interquartile range
MALERA: Malaria Eradication Research Agenda
MMP: Majete Malaria Project
mRDT: malaria rapid diagnostic test
ODK: open data kit
SP: sulfadoxine–pyrimethamine
WHO: World Health Organization
